# Harmonization study between three laboratories for expression of malaria vaccine clinical trial IgG antibody ELISA data in µg/mL

**DOI:** 10.1186/s12936-019-2935-3

**Published:** 2019-09-02

**Authors:** Geneviève M. Labbé, Kazutoyo Miura, Sarah E. Silk, Wenjuan Gu, James E. Moon, Jing Jin, Ruth O. Payne, Michael P. Fay, Sheetij Dutta, Carole A. Long, Simon J. Draper

**Affiliations:** 1 The Jenner Institute, University of Oxford, Old Road Campus Research Building, Oxford, OX3 7DQ UK; 2 Laboratory of Malaria and Vector Research, NIAID/NIH, Rockville, MD 20852 USA; 3 Biostatistics Research Branch, NIAID/NIH, Rockville, MD 20852 USA; 4 Military Malaria Research Program, Walter Reed Army Institute of Research, Silver Spring, MD 20910 USA

## Abstract

**Background:**

The ability to report vaccine-induced IgG responses in terms of µg/mL, as opposed arbitrary units (AU), enables a more informed interpretation of the magnitude of the immune response, and better comparison between vaccines targeting different antigens. However, these interpretations rely on the accuracy of the methodology, which is used to generate ELISA data in µg/mL. In a previous clinical trial of a vaccine targeting the apical membrane antigen 1 (AMA1) from *Plasmodium falciparum*, three laboratories (Oxford, NIH and WRAIR) reported ELISA data in µg/mL that were correlated but not concordant. This current study sought to harmonize the methodology used to generate a conversion factor (CF) for ELISA analysis of human anti-AMA1 IgG responses across the three laboratories.

**Methods:**

Purified IgG was distributed to the three laboratories and, following a set protocol provided by NIH, AMA1-specific human IgG was affinity purified. A new “harmonized CF” was generated by each laboratory using their in-house ELISA, and the original clinical trial ELISA data were re-analysed accordingly.

**Results:**

Statistical analysis showed that the data remained highly correlated across all three laboratories, although only Oxford and NIH were able to harmonize their CF for ELISA and generate concordant data.

**Conclusions:**

This study enabled two out of the three laboratories to harmonize their µg/mL readouts for the human anti-AMA1 IgG ELISA, but results reported from WRAIR are ~ twofold higher. Given the need to validate such information for each species and antigen of interest, it is important to bear in mind these likely differences when interpreting µg/mL ELISA data in the future.

## Background

Malaria, caused by *Plasmodium* parasites, continues to exert a huge burden on global public health, with over 200 million clinical cases annually and an estimated 435,000 related deaths [1]. The development of effective and durable vaccines thus remains a key public health priority to aid in the on-going global efforts to control and eliminate this disease [2].

Numerous stages of the parasite’s lifecycle are susceptible to vaccine-induced antibodies, including the liver-invasive sporozoite; the red blood cell (RBC)-invading merozoite; the infected erythrocyte (iRBC) which displays cell surface parasite-derived antigen; as well as the sexual-stage forms present in both the human host and mosquito vector [3]. These susceptibilities form the foundation of different vaccine strategies that seek to prevent malaria infection, disease or transmission via the induction of functional antibodies.

Central to these on-going efforts, and common to each strategy irrespective of life cycle stage target, is the need to down-select the best-performing vaccine candidates. Indeed such candidates can vary widely in terms of their vaccine delivery platform, formulation, target antigen(s) and immunogen design. Consequently, given the complexity of the malaria parasite, vaccine developers undertaking such studies are often posed with the important question: “which antigen is the “best” target for inclusion in a vaccine”? Answering this question is not trivial, and poses a number of challenges, especially when comparison of vaccine-induced polyclonal antibody (pAb) responses is required. Over recent years, researchers have, therefore, sought to address this problem through careful quantitative analysis of vaccine-induced pAb responses. Importantly, by measuring pAb using a mass concentration unit readout (typically µg/mL for serum IgG), as opposed to arbitrary units (AU) as most often reported, it becomes possible to undertake a more informed comparison of vaccine-induced pAb that target different antigens. Indeed, once measured in µg/mL, it can be established what concentration of antigen-specific IgG is required to afford a given level of activity using functional assays, or a given level of protection in vivo. Head-to-head comparative testing can thus establish which antigen performs “the best”—typically that is the antigen against which the lowest concentration of pAb is required to achieve the desired outcome.

However, although this concept is relatively straight-forward, it has proved challenging to develop methodologies to accurately measure polyclonal antigen-specific IgG responses raised by vaccination of humans or animals. This is because these responses form only a minor fraction of the total circulating IgG in plasma. Indeed in humans, for example, the average plasma concentration of total IgG is approximately 10 mg/mL, whilst vaccine-induced antigen-specific IgG responses are typically of the order of tens of µg/mL (although these can vary higher or lower by orders of magnitude dependent on the vaccine delivery strategy) [4–6]. To address this challenge, two main methodologies have been established in recent years, these are (i) the use of affinity-purified antigen-specific and species-specific IgG ELISA standards [7], or (ii) to undertake calibration-free concentration analysis (CFCA) on a Biacore system [8]. Both experimental approaches ultimately generate conversion factors (CF), that allow for the typical IgG ELISA AU readout to be converted into a mass concentration (routinely µg/mL). They also both require careful setup and assessment, and these studies need to be performed for each new vaccine antigen and target species IgG combination.

Apical membrane antigen 1 (AMA1) is a micronemal protein, expressed by the blood-stage malaria merozoite and a long-standing candidate vaccine antigen [9]. A range of different AMA1 based-vaccines have been assessed in Phase I/II clinical trials over the last decade. In 2016 a Phase I/IIa clinical trial (called VAC054) was reported, testing the recombinant AMA1 protein vaccine (FMP2.1) formulated in GlaxoSmithKline (GSK)’s Adjuvant System 01 (AS01). In this study, serum samples were analysed independently for anti-AMA1 human IgG antibodies by three different laboratories (at Oxford, NIH and WRAIR) using in-house ELISAs and recombinant AMA1 protein [10]. ELISA results in AU from each laboratory were then converted into μg/mL concentrations of anti-AMA1 IgG antibody using CFs that had been independently and historically established [6, 7, 11]. Although these results were highly correlated, they were not concordant (Additional file 1: Figure S1). Given the importance of ensuring accurate reporting of µg/mL concentrations, this present study aimed to harmonize the methodology used to generate the CF, and to assess whether this led to concordance of results between the three laboratories.

## Methods

### Human anti-AMA1 vaccine trial sera

The details of the VAC054 Phase I/IIa clinical trial have been published previously [10]. In brief, 15 healthy adult volunteers in the UK were immunized by intramuscular injection with a 50 µg dose of FMP2.1 protein vaccine formulated in AS01 adjuvant from GSK on days 0, 28 and 56. Twelve out of the 15 vaccinated volunteers then underwent blood-stage controlled human malaria infection (CHMI) 2 weeks after the final vaccination (on day 70) to assess vaccine efficacy. Following completion of this study, six 10 mL serum pools were prepared (using ELISA data from the VAC054 trial in order to cover a range of anti-AMA1 responses) and also including a pool of naïve (day 0) sera. Samples were labelled 1 to 6 in increasing order, with 1 being the naïve pool and 6 being the highest response pool. The VAC054 trial was registered with Clinicaltrials.gov (NCT02044198) and was conducted according to the principles of the current revision of the Declaration of Helsinki 2008 and in full conformity with the International Conference on Harmonization of Technical Requirements for Registration of Pharmaceuticals for Human Use guidelines for good clinical practice. The VAC054 study received ethical approval from the UK NHS Research Ethics Service (Oxfordshire Research Ethics Committee A, Ref 13/SC/0596), and the Western Institutional Review Board (WIRB) in the USA (Ref 20131985). The study was approved by the UK Medicines and Healthcare products Regulatory Agency (Ref 21584/0326/001-0001).

### Total IgG purification

Total IgGs were purified at NIH from each serum pool using protein G columns according to the manufacturer’s instructions (Pierce, Inc., Rockford, IL). The eluted fractions were immediately neutralized with Tris buffer (pH 9.0), dialyzed against RPMI 1640 medium (Thermo Fisher Scientific, UK), and concentrated with centrifugal filter devices to a concentration of 40 mg/mL. Total IgG from each pool was then divided into three fractions and distributed to the three laboratories participating in the study.

### AMA1-specific IgG purification

In each laboratory, AMA1-specific antibodies were isolated from total IgG aliquots using affinity purification columns, according to a protocol established by NIH.

First, the purification column was packed as follows: after adding the bottom frit to a disposable 10 mL column (Pierce) and 1 mL NHS-activated Sepharose slurry (product code 17-0906-01, GE Healthcare Life Sciences, Buckinghamshire, UK), 15 mL ice-cold 1 mM HCl was passed through the column to wash the slurry. Sepharose beads were resuspended with a mixture of 0.5 mL FMP2.1 AMA1 protein (provided by WRAIR) [12] at 1 mg/mL in PBS plus 0.25 mL coupling buffer (0.2 N NaHCO_3_, 0.5 N NaCl, pH 8.3). The column was sealed with parafilm and left rocking overnight at 4 °C. Next day, at room temperature (RT), the coupling buffer was drained and 5 mL blocking buffer (0.5 M ethanolamine, 0.5 M NaCl, pH 8.3) was passed through the column. After capping the bottom of the column, 10 mL blocking buffer was added; the top of the column was then capped and the column left rocking for 3 h at RT. The column was then centrifuged at 200 x*g* for 10 min, top frit was placed and blocking buffer drained. The column was then washed thrice with 2 mL blocking buffer, followed by three rinses with 2 mL wash buffer (0.1 M acetic acid, 0.5 M NaCl, pH 4), then another three rinses with blocking buffer. After capping the bottom of the column, 2 mL blocking buffer was added, left for 15 min, then drained. The column was again washed three times with 2 mL wash buffer, three times with 2 mL blocking buffer, and three times with 2 mL wash buffer. Next, 5 mL binding buffer (50 mM Na_2_HPO_4_, pH 7.0) was passed through the column, followed by 5 mL 20% ethanol, of which only about 3 mL was drained before capping the column for storage at 4 °C.

Subsequently, affinity adsorption was performed as follows: total IgG (0.5–0.9 mL of each sample, 40 mg/mL stocks) was mixed with 9 volumes of room-temperature binding buffer. The column was washed with 5 mL room-temperature binding buffer and 5 mL room-temperature elution buffer (0.1 M glycine, pH 2.7), and equilibrated with 5 mL binding buffer. After placing a clean tube to collect flow-through, the first sample (naïve pool) was applied to the column, and flow-through re-applied 4 extra times to ensure complete binding. The column was then washed with 10 mL binding buffer. AMA1-specific IgG were eluted with 5 mL elution buffer into a 15 mL tube containing 300 µL of 1 M Tris, pH 9.0. Upon immediate mixing, IgG were stored at 4 °C. The column was then washed with 5 mL elution buffer, re-equilibrated with 5 mL binding buffer, and the second sample was applied as described above. The same procedure was followed for samples 3, 4, 5 and 6, in this order. The column was then rinsed and stored in 20% ethanol at 4 °C.

Subsequently, AMA1-specific IgG were dialyzed against RPMI 1640 and concentrated to 50–100 µL final product: for each sample, 4 mL of elution fraction was applied to an Amicon Ultra-4 Centrifugal Filter Unit (30 kDa membrane EMD Millipore, Cat # UFC803024) and spun at 3000–4000×*g* at 4 °C for 10 min. After discarding the flow-through, the left-over 1 mL elution fraction was added to the filter and spun for 20-30 min until 100–150 µL remained above the filter. Flow-through was discarded, and RPMI 1640 medium was used to fill the device up to 4 mL, mixed with the sample, and centrifuged until 100–150 µL remained above the filter. RPMI 1640 was again added to 4 mL, mixed and centrifuged, aiming for a final volume of 50–100 µL. Concentrated IgG was then transferred into a 0.5 mL sterile tube and stored at 4 °C. Finally, the concentration of AMA1-specific IgG was measured by absorbance at 280 nm using NanoDrop (NanoDrop, Wilmington, DE, USA).

### AMA1 proteins and ELISA methods

For each eluted AMA1-specific fraction, the anti-AMA1 antibody response in AU was measured by each laboratory, using the same ELISA protocol as they had used for the analyses of the VAC054 clinical trial [10]; i.e., each laboratory determined AUs using their own protocol and AMA1 protein, unless specified otherwise. The differences in the human anti-AMA1 IgG ELISA procedures in the three laboratories are summarized in Additional file 1: Table S1. The WRAIR protein, FMP2.1, is a recombinant AMA1 from the 3D7 clone of *Plasmodium falciparum* produced in and purified from *Escherichia coli*, carrying hexa-histidine (His6) tags both N- and C-terminally [12]. The NIH protein, AMA1-3D7 (lot MV#1183), was produced in *Pichia pastoris* and carries a C-terminal His6-tag [13]. The Oxford protein, PfAMA1 (3D7).BAP.HIS (lot P0146), was produced in mammalian HEK293 cells as previously described [6]. All proteins included the full AMA1 ectodomain.

### AMA1 protein comparison by ELISA at Oxford

Three Nunc-immuno maxisorp plates were coated with 2 μg/mL Oxford, NIH, or WRAIR AMA1 protein in DPBS. The same independent standard curve (10 points) was run on each plate in duplicate, using a previously described high-titer anti-AMA1 human serum reference sample [14]. Otherwise, the same ELISA method for this experiment was performed on all three plates in parallel: plates were left at RT overnight, then washed 6 times with PBS containing 0.05% Tween 20 (PBS/T) and blocked for 1 h with Casein block solution (Pierce). After another wash step, samples were added to each plate for 2 h. Plates were washed again and alkaline phosphatase-conjugated goat anti-human IgG (γ-chain) (Sigma) diluted 1:1000 in Casein block solution was added for 1 h, before development with *p*-nitrophenylphosphate substrate (Sigma) diluted in diethanolamine buffer (Thermo Fisher Scientific). Optical density at 405 nm (OD_405_) was read using a microplate reader (Biotek) and Gen5 v1 software.

### Statistical analysis

To determine the new, harmonized conversion factor (CF; mass concentration of antibody which gives 1 AU), a linear regression was performed using AU and protein concentration of AMA1-specific IgGs generated in this study (calibration data set). Subsequently, AU values for 12 dC-1 (day 69, the day before CHMI) samples of VAC054 study measured in each laboratory (reported previously [10]) were multiplied by the laboratory-specific CF, in order to transform the antibody level of each sample tested in each laboratory into mass concentration (MC data set). The MC data sets between two laboratories were used to determine the concordance correlation coefficient (CCC), more specifically RMAC version of the concordance coefficient, as described previously [15]. To determine confidence intervals for the CCC, best-fit slope, and Pearson’s correlation coefficient of MC data sets among different laboratories, considering errors in both CF and AU determinations, a bootstrap method was used. Specifically, the following steps were performed: (i) choose a random sample with replacement from the calibration data set, calculate CF for each laboratory using a linear regression; (ii) choose a random sample with replacement from the MC data set except replace the CF originally used with the CF determined from step (i) for each laboratory. In step (ii), paired MC data samples were selected for all labs. For example, if sample # 1, 2, 2, 4, 5 and 6 of the MC data set were randomly selected from a laboratory, then sample # 1, 2, 2, 4, 5 and 6 from the other two laboratories were used for analysis; (iii) calculate pairwise CCC, best-fit slope, and Pearson’s correlation coefficient using the step ii data set; (iv) repeat steps (i)–(iii) 10,000 times, then use the 95% BCa bootstrap interval from those replicates [16]. Confidence intervals were inverted to get *P* values.

In separate analyses, to compare the MC data set from the three laboratories, a Friedman test followed by Wilcoxon signed rank test was used. To compare OD values of the serially diluted anti-AMA1-3D7 anti-serum pool tested in a single laboratory (Oxford), but against three different proteins, linear regression analyses were performed, and slope, R^2^ and *P* value (whether the slope is significantly different from 1) were calculated.

Analyses were performed using GraphPad Prism version 7.03 for Windows (GraphPad Software Inc., California, USA) and R version 3.3.2. A *P* value < 0.05 was considered significant.

## Results

Six pools of 10 mL serum samples were generated in Oxford from vaccinees in the VAC054 trial. Samples were selected from various time-points and volunteers so as to generate a spread of anti-AMA1 IgG responses across the six different pools. One of the six pools was pre-immunization (day 0) serum. The sera were provided to NIH who performed protein G purification of each sample, prior to distribution of one aliquot of each purified IgG to Oxford and WRAIR.

**Fig. 1:**
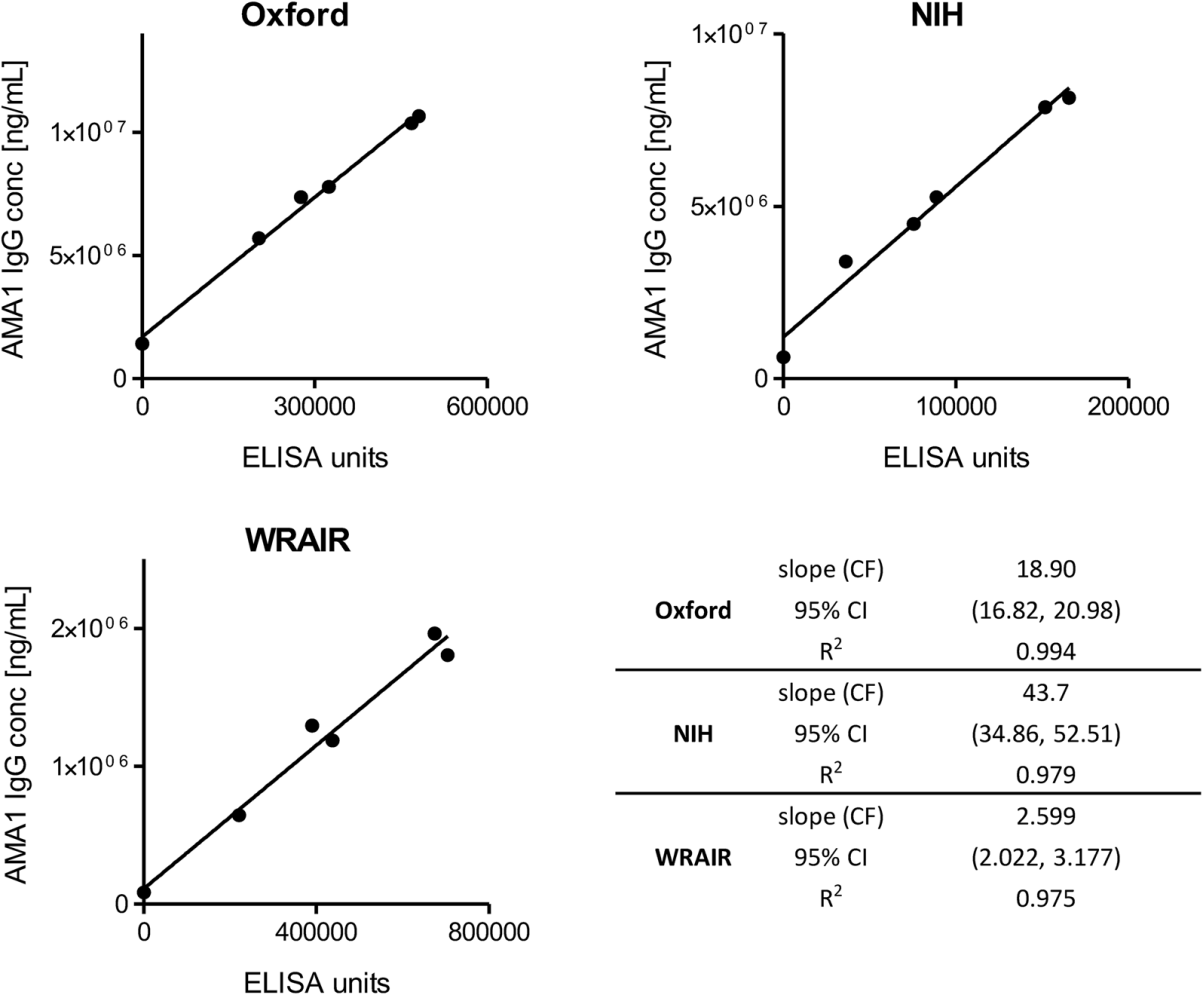
Conversion factors determined using the harmonized method. Six total IgG samples from the VAC054 trial were divided amongst the three laboratories and used to determine conversion factors. AMA1-specific IgG was affinity-purified using the same protocol and FMP2.1 protein coupled to purification columns. The concentration of thus affinity-purified AMA1-specific IgG in ng/mL was plotted against ELISA AU obtained using in-house ELISA protocols with in-house AMA1 protein. The slope of each linear regression line was used as the “harmonized conversion factor” (CF)

**Fig. 2:**
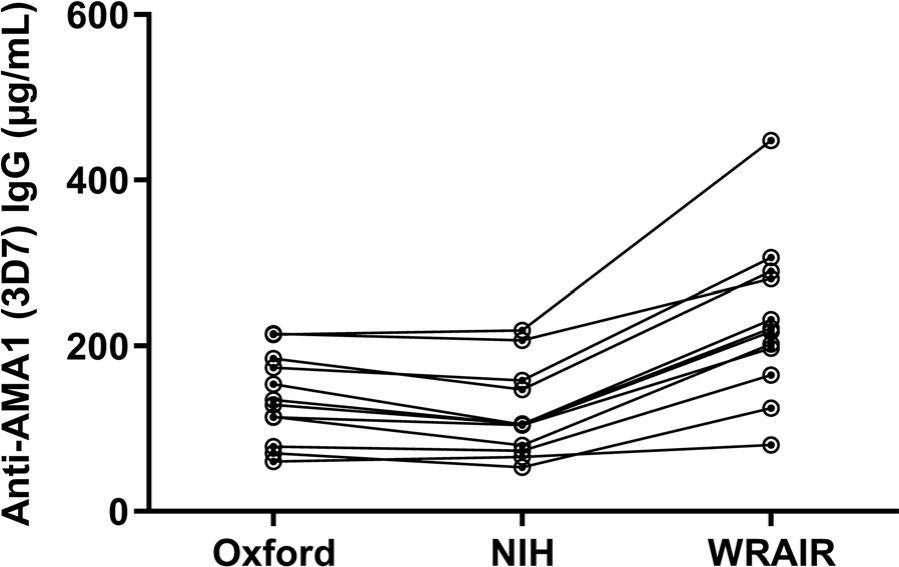
VAC054 ELISA data using harmonized conversion factors. The original VAC054 ELISA data for the day before CHMI (dC-1) were re-analysed using the new harmonized CFs. Twelve vaccinees underwent CHMI and their anti-AMA1 serum IgG responses are reported. Individual responses are shown with a connecting line for each sample

Each laboratory subsequently processed and analysed each sample according to a set protocol provided by NIH. Initially, AMA1-specific IgG were affinity-purified from each sample using FMP2.1 protein, which was used in the VAC054 trial, coupled to columns as described in the Methods section, prior to buffer exchange and concentration. The concentration of each affinity-purified AMA1-specific IgG was measured by Nanodrop and analysed by the in-house ELISA protocol at each laboratory (Additional file 1: Table S1), using in-house AMA1 protein as a coating antigen for ELISA. IgG concentration was then plotted against ELISA AU and the slope of each linear regression line was used as the new “harmonized conversion factor (CF)” (Fig. 1). Subsequently, these harmonized CFs were used to re-analyse the original VAC054 ELISA data (Additional file 1: Fig. S1), thus reporting the amount of AMA1-specific human IgG per ELISA AU in µg/mL (Fig. 2). In the analysis, a fixed harmonized CF (i.e. the best estimate value) for each laboratory was utilized. The median [range] of the 12 responses were: Oxford = 132 [60–215] µg/mL; NIH = 105 [54–219] µg/mL; and WRAIR = 219 [80–448] µg/mL. Notably, when antibody levels in the µg/mL scale were compared from the three laboratories, there was a significant difference among the three (*P *< 0.0001; Friedman test), and the differences of all three comparisons were significant (*P *= 0.003 for Oxford vs. NIH; *P* = 0.0005 for WRAIR vs. Oxford or NIH).

Since the above statistical analysis did not account for the error in the harmonized CF determinations (see Fig. 1 for the 95% CI for each CF), further bootstrap analysis was performed to compare the data from the three laboratories. The results indicated that the new ELISA data in mass concentration between WRAIR and both Oxford and NIH remained non-concordant when applying the harmonized CFs (Table 1), despite all three datasets showing a strong correlation. However, there was strong concordance of ELISA data (n = 12) between NIH and Oxford laboratories when applying their harmonized CFs (CCC = 0.898 [95% CI 0.580, 0.971], Table 1). Thus overall, this study had enabled two out of the three laboratories to harmonize their µg/mL readouts for the human anti-AMA1 IgG ELISA.

**Table 1:** Statistical analysis of harmonized data

Laboratory	Oxford vs. WRAIR	Oxford vs. NIH	NIH vs. WRAIR
Concordance			
Concordance coefficient (CC)	0.2987	0.8979	0.1693
Bootstrap 95% CI	(− 0.2105, 0.5635)	(0.5796, 0.9713)	(− 0.4382, 0.5359)
*P* value	0.2398	0.0064	0.6441
Linear regression			
Slope	0.5034	0.9381	0.5095
Bootstrap 95% CI	(0.3663, 0.8545)	(0.7418, 1.3191)	(0.3490, 0.8517)
*P* value^a^	0.0096	0.5933	0.0148
Correlation			
Pearson’s correlation coefficient	0.9036	0.9489	0.904
Bootstrap 95% CI	(0.8098, 0.9820)	(0.8975, 0.9850)	(0.8023, 0.9927)
*P* value	0.0082	0.0002	0.0082

Bootstrap analysis of the new ELISA data in mass concentration scale was undertaken (n = 12) to assess for concordance. Linear regression and correlation analyses are also shown. The 95% confidence intervals (CI) were calculated using percentiles from 10,000 bootstraps

^a^Testing the null hypothesis of slope = 1

**Fig. 3:**
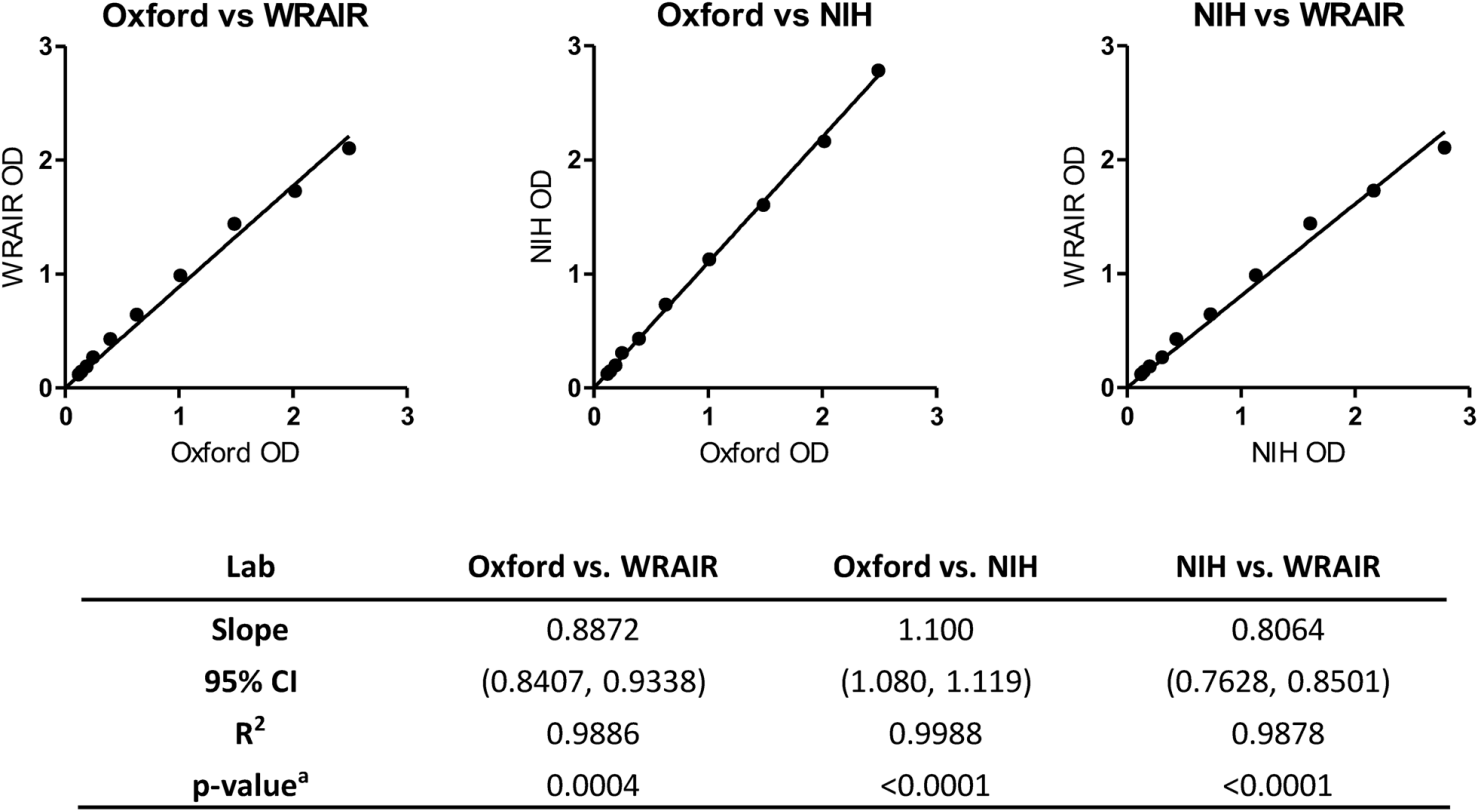
ELISA comparison of AMA1 proteins used by Oxford, NIH and WRAIR. The same anti-AMA1 antiserum was assessed by ELISA using identical methodology (Oxford method) on plates coated with AMA1 protein provided by each of the three laboratories (Oxford, NIH and WRAIR). The test sample was plated in a dilution series, and the points show the mean of duplicate readings. Linear regression analysis of OD_405_ nm readings is shown. The linear regression line was forced to go through point (X = 0, Y = 0). ^a^Testing the null hypothesis of slope = 1

To uncover the mechanism of discrepancy, the three recombinant AMA1 proteins used by the different laboratories were compared side-by-side in an ELISA conducted at Oxford. At NIH, the protein was produced in *Pichia pastoris* yeast [13]; at Oxford, AMA1 protein was produced in a mammalian HEK293 cell system [6]; and at WRAIR, the FMP2.1 AMA1 protein was produced in *Escherichia coli* [12]. The same test sample was assessed using identical methodology on plates coated with AMA1 protein provided by each of the three laboratories. Analysis by linear regression highlighted small, but significant, differences (Fig. 3). In particular, for the same antibody samples tested, the WRAIR coat protein generally gave lower OD_405_ readings compared to NIH/Oxford and it appeared that the two highest data points on the WRAIR OD axis were drifting towards saturation at around OD_405_ = 2 (Fig. 3).

## Discussion

This current study sought to assess whether the methodology used to generate a CF for ELISA analysis of human anti-AMA1 IgG responses across three laboratories could be harmonized. Historical data from the VAC054 trial showed the data from Oxford, NIH and WRAIR were correlated but not concordant, suggesting a discrepancy in the CFs used to convert ELISA AU to µg/mL. The ability to report vaccine-induced IgG responses in terms of µg/mL, as opposed AU, is highly informative, enabling i) a better understanding of the magnitude of the immune response, and ii) better comparison between vaccines targeting different antigens. However, these interpretations rely on the accuracy of the CF which is used to generate ELISA data in µg/mL, and it is important to establish whether independent laboratories can achieve concordant data.

Six serum pools were initially prepared from the VAC054 clinical trial in Oxford and the total IgGs were then purified at NIH, before distribution to the three laboratories participating in the study. Using a set protocol provided by NIH, AMA1-specific human IgG were independently affinity-purified from each sample using FMP2.1 protein provided by WRAIR. A new “harmonized CF” was generated by each laboratory using their in-house AMA1 protein as a coating antigen for ELISA and the original VAC054 clinical trial ELISA data were re-analysed accordingly. As shown in Fig. 1, the protocol of “harmonized CF” determination used in this study provided a strong linear correlation between ELISA units and antigen-specific protein concentration in each of the three laboratories (R^2^ > 0.975). The results thus indicated the antigen-specific IgG affinity purification protocol could provide the accurate estimate of CFs in different laboratories. However, compared to CFCA for example, this method requires larger amounts of antigen and antisera. Therefore, the best methodology to determine a CF should be selected depending on the goal of the study and availability of test materials. The reproducibility between laboratories of other methods, such as CFCA, has not yet been assessed, although one small analysis from Oxford showed comparable results between the two methods [6].

The initial analysis was undertaken as traditionally done in the literature—using the defined point estimate of the harmonized CF to convert AU data to µg/mL (Fig. 1). In this case, the median and range of the datasets from Oxford and NIH were very comparable, however, the dataset from WRAIR showed a roughly twofold higher median and twofold greater range (Fig. 2). Statistical analysis by Friedman test followed by Wilcoxon signed rank tests, however, also reported that the datasets were all significantly different. The significant difference between the Oxford and NIH datasets was due to systematically smaller (but not by much, see Fig. 2 and Additional file 1: Fig. S1) measurements from the NIH. This result did not account for the size of the differences or the variability in estimates of the harmonized CFs. When those two effects were accounted for by the subsequent bootstrap analysis of the data (Table 1), there was strong concordance of ELISA data between the NIH and Oxford laboratories when applying their harmonized CFs (CCC = 0.898 [95% CI 0.580, 0.971]). On the contrary, the dataset from WRAIR had much smaller and non-significant estimates of concordance, but was still closely correlated with the datasets from NIH and Oxford.

Although this enabled two out of the three laboratories to harmonize their µg/mL readouts for the human anti-AMA1 IgG ELISA, the lack of strong concordance between the NIH/Oxford datasets and the data from WRAIR was not explained. Small, but significant, differences were noted in the OD_405_ response of the three AMA1 proteins used for in-house ELISAs which potentially contributed to this result (Fig. 3). These proteins were made historically and have small differences in terms of amino acid sequences and each is made in a different expression platform (bacterial, yeast *versus* mammalian cells). The difference in ODs among different proteins (Fig. 3) could be partially explained by the characteristics of them (binding capacity to the ELISA plates, glycosylation modification, etc.). However, the difference in OD values among the three AMA1 proteins was only 10–20% (the best-fit slopes were 0.806 to 1.100), which might be too small to explain the ~ twofold difference in µg/mL readouts (Table 1). In addition, WRAIR protein on the plate may offer a lower dynamic range at higher antibody concentrations, as lower resolution of some data points was also observed along the line of best fit in Fig. 1. The other factors which might contribute to the discrepancy (at least in part) could be the differences in batches of commercial reagents and ELISA protocols used at the different sites. It would be ideal to determine whether the ~ twofold difference in µg/mL readouts among three laboratories is a reproducible phenomenon and to uncover the mechanism of this difference experimentally. However, human anti-AMA1 antibodies from the study are very limited and it is now difficult to perform such additional studies. For now, it remains that the human anti-AMA1 IgG ELISA readout in µg/mL from WRAIR is approximately twofold higher than that from Oxford and NIH, and thus this difference should be held in mind when interpreting clinical trial ELISA data in the literature.

## Conclusion

This study attempted to harmonize CFs to achieve concordant ELISA measurements in µg/mL for one species (human) and antigen (AMA1) between three independent laboratories using the antigen-specific IgG affinity purification methodology. Although successful across two laboratories, there was not strong concordance across all three, despite achieving highly correlated ELISA data. The reasons for these differences remain to be fully determined, however our study underlines the difficulties associated with determining µg/mL readouts for antigen-specific ELISAs. This study suggests that determining the harmonized CFs may not be sufficient to obtain concordant results from different laboratories. More stringent harmonization (e.g., sharing the same protein and using the same protocol for each step) would be required. While that is the ideal scenario, given the need to validate such procedures for each species and antigen of interest, and the possibility that the studies could be conducted with multiple different antigens, it might be practically challenging to perform all studies in a “harmonized” fashion. Therefore, it is important to bear in mind these likely differences when interpreting data from different studies in the future.

## Supplementary information

**Additional file 1:Figure S1.** VAC054 dC-1 ELISA results, converted to anti-AMA1 (3D7) IgG (μg/mL) using independent vs harmonized conversion factors. **Table S1.** ELISA methods in the three laboratories.

## Data Availability

The datasets arising and/or analysed during the current study are available from the corresponding author on reasonable request.

## Abbreviations

AMA1: apical membrane antigen 1
AS01: Adjuvant System 01
AU: arbitrary units
CCC: concordance correlation coefficient
CF: conversion factor
CFCA: calibration-free concentration analysis
CHMI: controlled human malaria infection
CI: confidence intervals
GSK: GlaxoSmithKline
His6: hexa-histidine
iRBC: infected red blood cell/erythrocyte
pAb: polyclonal antibody
PBS/T: PBS containing 0.05% Tween 20
RT: room temperature
