# Supplementary material for: Harmonization study between three laboratories for expression of malaria vaccine clinical trial IgG antibody ELISA data in µg/mL
Source: Malar J. 2019 Sep 2;18:300. doi: 10.1186/s12936-019-2935-3 (PMC6721210; doi:10.1186/s12936-019-2935-3)
Supplement: Supplementary file 1 — Additional file 1:Figure S1. VAC054 dC-1 ELISA results, converted to anti-AMA1 (3D7) IgG (μg/mL) using independent vs harmonized conversion factors. Table S1. ELISA methods in the three laboratories. [file 12936_2019_2935_MOESM1_ESM.docx]

# Additional File

## Figure S1: VAC054 dC-1 ELISA results, converted to anti-AMA1 (3D7) IgG (μg/mL) using independent vs harmonized conversion factors.

Spearman’s correlation and linear regression analyses of ELISA data (n = 12) from the three laboratories when applying independently established, versus harmonized conversion factors. Data from independently established conversion factors, showing correlated not concordant results, are included here for clarity but were previously published in [1]. Reference lines of equality are plotted as a dashed line.

|  | **Oxford** | **WRAIR** | **NIH** |
| --- | --- | --- | --- |
| **Plate Coating** | 2 µg/mL of AMA1  RT overnight | 1 µg/mL of AMA1  4 °C overnight | 10 µg/mL of AMA1  4 °C overnight |
| **Blocking** | Casein block solution RT for 1 h | Casein block solution  RT for 1-2 h | 5 % milk  RT for 2 h |
| **Primary Antibody** | RT for 2 h | RT for 2 h | RT for 2 h |
| **Secondary Antibody** | AP-labelled goat anti-human IgG (γ-chain) (Sigma A3187)  RT for 1 h | Peroxidase-Labelled antibody to human IgG(γ-chain) (KPL/074-1002)  RT for 1 h | AP-labelled goat anti-human IgG (H+L) (Kirkegaard & Perry Labs, 075-106)  RT for 2 h |
| **OD Reading** | 405 nm | 414 nm | 405/650 nm |
| **Convert OD to AU** | AU of a test sample is determined relative to the serially diluted ELISA standard tested in the same plate | AU of a test sample is determined relative to the serially diluted ELISA standard tested in the same plate | AU of a test sample is determined relative to the serially diluted ELISA standard tested in the same plate |
| **Reference(s)** | [2, 3] | [4] | [5] |

## Table S1: ELISA methods in the three laboratories.

# Competing Interests

SJD is a named inventor on patent applications relating to malaria vaccines and immunization regimens. SD has a patent on the FMP2.1 vaccine antigen.

# Additional References

1. Payne RO, Milne KH, Elias SC, Edwards NJ, Douglas AD, Brown RE, Silk SE, Biswas S, Miura K, Roberts R, et al: **Demonstration of the Blood-Stage Controlled Human Malaria Infection Model to Assess Efficacy of the Plasmodium falciparum AMA1 Vaccine FMP2.1/AS01.** *J Infect Dis* 2016, **213:**1743-1751.

2. Hodgson SH, Choudhary P, Elias SC, Milne KH, Rampling TW, Biswas S, Poulton ID, Miura K, Douglas AD, Alanine DG, et al: **Combining Viral Vectored and Protein-in-adjuvant Vaccines Against the Blood-stage Malaria Antigen AMA1: Report on a Phase 1a Clinical Trial.** *Mol Ther* 2014, **22:**2142-2154.

3. Sheehy SH, Duncan CJ, Elias SC, Biswas S, Collins KA, O'Hara GA, Halstead FD, Ewer KJ, Mahungu T, Spencer AJ, et al: **Phase Ia Clinical Evaluation of the Safety and Immunogenicity of the Plasmodium falciparum Blood-Stage Antigen AMA1 in ChAd63 and MVA Vaccine Vectors.** *PLoS One* 2012, **7:**e31208.

4. Spring MD, Cummings JF, Ockenhouse CF, Dutta S, Reidler R, Angov E, Bergmann-Leitner E, Stewart VA, Bittner S, Juompan L, et al: **Phase 1/2a study of the malaria vaccine candidate apical membrane antigen-1 (AMA-1) administered in adjuvant system AS01B or AS02A.** *PLoS ONE* 2009, **4:**e5254.

5. Miura K, Orcutt AC, Muratova OV, Miller LH, Saul A, Long CA: **Development and characterization of a standardized ELISA including a reference serum on each plate to detect antibodies induced by experimental malaria vaccines.** *Vaccine* 2008, **26:**193-200.
